# Rocker-sole footwear versus prefabricated foot orthoses for the treatment of pain associated with first metatarsophalangeal joint osteoarthritis: study protocol for a randomised trial

**DOI:** 10.1186/1471-2474-15-86

**Published:** 2014-03-15

**Authors:** Hylton B Menz, Pazit Levinger, Jade M Tan, Maria Auhl, Edward Roddy, Shannon E Munteanu

**Affiliations:** 1 Lower Extremity and Gait Studies Program, Faculty of Health Sciences, La Trobe University, Bundoora 3086, Victoria, Australia; 2 Department of Podiatry, Faculty of Health Sciences, La Trobe University, Bundoora 3086, Victoria, Australia; 3 Institute of Sport, Exercise and Active Living, Victoria University, Melbourne 8001, Victoria, Australia; 4 Arthritis Research UK Primary Care Centre, Research Institute for Primary Care and Health Sciences, Keele University, Staffordshire ST5 5BG, UK

## Abstract

**Background:**

Osteoarthritis affecting the first metatarsophalangeal joint of the foot is a common condition which results in pain, stiffness and impaired ambulation. Footwear modifications and foot orthoses are widely used in clinical practice to treat this condition, but their effectiveness has not been rigorously evaluated. This article describes the design of a randomised trial comparing the effectiveness of rocker-sole footwear and individualised prefabricated foot orthoses in reducing pain associated with first metatarsophalangeal joint osteoarthritis.

**Methods:**

Eighty people with first metatarsophalangeal joint osteoarthritis will be randomly allocated to receive either a pair of rocker-sole shoes (MBT® Matwa, Masai Barefoot Technology, Switzerland) or a pair of individualised, prefabricated foot orthoses (Vasyli Customs, Vasyli Medical™, Queensland, Australia). At baseline, the biomechanical effects of the interventions will be examined using a wireless wearable sensor motion analysis system (LEGSys™, BioSensics, Boston, MA, USA) and an in-shoe plantar pressure system (Pedar®, Novel GmbH, Munich, Germany). The primary outcome measure will be the pain subscale of the Foot Health Status Questionnaire (FHSQ), measured at baseline and 4, 8 and 12 weeks. Secondary outcome measures will include the function, footwear and general foot health subscales of the FHSQ, severity of pain and stiffness at the first metatarsophalangeal joint (measured using 100 mm visual analog scales), global change in symptoms (using a 15-point Likert scale), health status (using the Short-Form-12® Version 2.0 questionnaire), use of rescue medication and co-interventions to relieve pain, the frequency and type of self-reported adverse events and physical activity levels (using the Incidental and Planned Activity Questionnaire). Data will be analysed using the intention to treat principle.

**Discussion:**

This study is the first randomised trial to compare the effectiveness of rocker-sole footwear and individualised prefabricated foot orthoses in reducing pain associated with osteoarthritis of the first metatarsophalangeal joint, and only the third randomised trial ever conducted for this condition. The study has been pragmatically designed to ensure that the findings can be implemented into clinical practice if the interventions are found to be effective, and the baseline biomechanical analysis will provide useful insights into their mechanism of action.

**Trial registration:**

Australian New Zealand Clinical Trials Registry: ACTRN12613001245785

## Background

Osteoarthritis (OA) is the leading cause of chronic pain and disability in Australia. In 2013, it was estimated that 1.9 million people in Australia had OA, and that this figure is expected to increase to 3 million people by 2032 [1]. Although the knee is the most commonly affected lower limb region, foot involvement is also common. The most commonly affected region of the foot is the first metatarsophalangeal joint (MTPJ), with radiographic changes evident in 12 to 35% of people aged over 35 years [2]. The population prevalence of symptomatic radiographic first MTPJ OA (i.e. both radiographic changes and associated symptoms) in people aged over 50 years has recently been estimated as 7.8%, with a higher prevalence observed in women, older people, and those from lower socio-economic classes [3].

People with first MTPJ OA typically present with symptoms of pain and stiffness in their big toe joint that increase with activity. Because the first MTPJ plays an important role in the transfer of the body during the propulsive phase of gait, people with first MTPJ OA will often adopt an apropulsive walking pattern with a shortened step length and longer stance phase duration [4], accompanied by excessive knee and hip flexion or hip circumduction to assist the transfer of the swing limb [5] and decreased loading of the first MTPJ [6]. In our recently completed comparison of 43 cases and 43 controls, we found that symptomatic first MTPJ OA was associated with a statistically significant reduction in all subscales of the Foot Health Status Questionnaire and the physical function and subscale of the Medical Outcomes Study Short Form 36 questionnaire [7]. In addition, Abhishek *et al*. have reported that great toe pain is associated with lower scores on the health satisfaction and psychological domains of the World Health Organization Quality of Life questionnaire [8], and a recent population-based study found that 72% of people with symptomatic first MTPJ OA report disabling symptoms [3].

Many patients with first MTPJ OA seek surgical treatment for their condition. A clinical audit of 785 cases of foot surgery in Australia between July 1995 and June 1996 revealed surgery for this condition to be the fourth most common procedure performed [9]. Similarly, a population-based study of 6,956 inpatients in Sweden [10] indicated that these procedures accounted for 15% of all foot and ankle procedures. Our recent analysis of the Medicare Benefits Schedule database indicated that between 1997 and 2006, over 46,000 private sector surgical procedures for treatment of first MTPJ disorders (hallux valgus and hallux rigidus) were subsidised under Medicare, at a direct cost of approximately $2 M per year [11]. These costs, however, are an underestimate of the total economic impact of the condition. The 2013 Deloitte Access Economics analysis indicated that treatment expenses for OA accounted for only 17% of the total cost of the disease, the remaining costs being attributed to burden of disease and productivity losses [1].

The aetiology of first MTPJ OA is not fully understood. Increased age, previous trauma, family history and a flat foot are commonly reported as contributing factors in the literature, but few studies have been undertaken to examine this in detail [5]. There is, however, evidence that certain structural characteristics of the foot are more common in those with first MTPJ OA compared to controls. Our recent systematic review concluded that people with first MTPJ OA were more likely to exhibit a dorsiflexed first metatarsal relative to the second metatarsal, a plantarflexed forefoot on the rearfoot, reduced first MTPJ joint range of motion, a longer proximal phalanx, distal phalanx, medial and lateral sesamoids, and a wider first metatarsal and proximal phalanx [12]. Although temporal relationships cannot be inferred from such studies, it has been hypothesised that as a result of this altered foot structure, the first metatarsal is less able to plantarflex during propulsion to allow the proximal phalanx to dorsally rotate on the first metatarsal head, resulting in dorsal joint compression of the first MTPJ. Over time, this process results in cartilage damage, exposure of subchondral bone, juxta-articular sclerosis, and the formation of a dorsal osteophyte [5].

Conservative treatment of first MTPJ OA involves measures to obtain pain relief (including anti-inflammatory medications and intra-articular injections), physical therapy to maintain range of motion, and footwear and foot orthoses to modify foot function. Surgical management may involve removal of the dorsal exostosis, insertion of a joint implant, or, in advanced cases, fusion of the first MTPJ [13]. However, despite the high prevalence of first MTPJ OA and its impact on quality of life, very few of these treatments have been rigorously evaluated. Furthermore, in patients with more advanced disease, post-surgical complications such as transfer lesions, forefoot pain, malunion and interphalangeal OA are common [14]. Our recent systematic review of interventions for first MTPJ OA [15] revealed only one low quality randomised trial evaluating the effectiveness of two different physical therapy programs in 20 participants [16]. Following the publication of this review, we completed a randomised controlled trial which found that intra-articular viscosupplementation (hylan G-F20) was no more effective than a placebo (sterile saline) for this condition [17]. Clearly, there is a need for additional well-designed trials into non-surgical interventions for the treatment of first MTPJ OA.

The pain associated with first MTPJ OA generally occurs during the propulsive phase of gait when the proximal phalanx is compressed against the dorsal osteophyte on the first metatarsal head. Therefore, ameliorating the need for the first MTPJ to dorsiflex has the potential to reduce joint compression and alleviate pain when walking. Clinically, this can be achieved using a footwear modification known as a rocker-sole, in which the sole of the shoe is curved (at the forefoot, rearfoot, or both) [18]. The aim of this modification is to allow the body’s centre of mass to “roll over” the base of support, reducing the need for foot and ankle dorsiflexion and subsequently decreasing the loads placed on the forefoot. Biomechanical studies have confirmed this proposed mechanism, indicating that rocker-sole shoes reduce sagittal plane motion of the forefoot [19] and ankle [19-22], reduce forefoot plantar pressures [22,23] and reduce first MTPJ dorsiflexion [24] when walking. Such a change in biomechanics may be therapeutically beneficial in those with first MTPJ OA by reducing compression at the first MTPJ. Although no controlled studies have been performed, uncontrolled reports suggest that rocker-sole shoes are effective in the management of first MTPJ pain, and may in some cases prevent the need for surgery [14,25,26].

In both clinical practice and research, the main barrier to the use of therapeutic footwear is concern regarding aesthetics, and indeed, adherence to therapeutic footwear in people with diabetes or rheumatoid arthritis has been reported to be as low as 22% [27-29]. However, the recent popularity of so-called “physiological” or “toning” footwear, pioneered by the Swiss company Masai Barefoot Technology (MBT), has made the rocker-sole shoe more fashionable. It is therefore likely that acceptability and adherence to this intervention will be much greater than that associated with the relatively unattractive medical grade footwear that has been used for this purpose previously. Indeed, a recent trial of MBT shoes for knee OA reported a drop-out rate of only 2% over 12 weeks [30].

In addition to rocker-sole shoes, first MTPJ OA is often managed with prefabricated or customised foot orthoses. Foot orthoses are thought to decrease pain associated with this condition by allowing the first metatarsal to achieve sufficient plantarflexion in preparation for propulsion, thereby minimising joint compression [31]. Although widely used and recommended in clinical practice guidelines [32], evidence to support the effectiveness of this approach is limited to case reports [26,33,34] and one recent case series study, which demonstrated a clinically significant reduction in pain over 24 weeks in 32 participants [35].

Given the prevalence and impact of first MTPJ OA, the lack of evidence for existing interventions, and the preliminary biomechanical and anecdotal evidence supporting the use of rocker-sole shoes and prefabricated foot orthoses, there is a need to conduct a rigorous randomised trial to evaluate which of these non-invasive treatments is most effective.

## Methods

The trial has been registered on the Australian New Zealand Clinical Trials Registry (ACTRN12613001245785).

### Ethical approval

The La Trobe University Human Ethics Committee has provided ethical approval (number 13–003). All participants will provide written informed consent prior to enrolment. Ethical standards will adhere to the National Health and Medical Research Council (NHMRC) National Statement [36] and the World Medical Association's Declaration of Helsinki [37]. Publications associated with the trial will be reported according to the Consolidated Standards of Reporting Trials (CONSORT) 2010 Statement [38,39].

## Design

**Figure 1:**
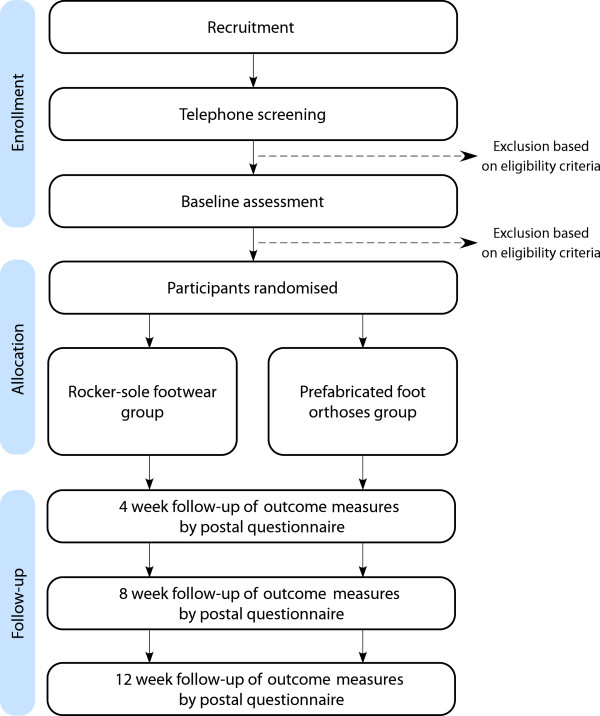
Trial profile.

The study design is a parallel-group randomised trial with 12 week follow-up (see Figure 1), comparing two interventions: commercially available rocker-sole footwear (MBT® Matwa, Masai Barefoot Technology, Switzerland) *versus* prefabricated foot orthoses (Vasyli Customs, Vasyli Medical™, Queensland, Australia). The study has been designed using the principles described by Osteoarthritis Research Society International Clinical Trials Task Force guidelines [40]. Permuted block randomisation with random block sizes, stratified by sex, will be undertaken using an interactive voice response telephone service provided by the NHMRC Clinical Trials Centre at the University of Sydney, New South Wales, Australia to ensure allocation concealment. Participants will be informed that they will receive either the rocker-sole footwear or the foot orthoses (i.e. they will not be blinded to their group allocation). Due to the nature of the intervention, research staff administering the treatments cannot be blinded to group allocation. However, follow-up assessment of outcome measures will be via self-completion questionnaires returned by mail, and those entering outcome measure data and conducting statistical analyses will be blinded.

### Participant recruitment, screening and eligibility criteria

To be included in the study, participants must:

(i) be aged at least 18 years;

(ii) report having pain in the first MTPJ on most days for at least 12 weeks;

(iii) report having pain rated at least 20 mm on a 100 mm visual analogue scale (VAS);

(iv) have less than 64 degrees of dorsiflexion range of motion of the first MTPJ [41];

(v) have pain upon palpation of the dorsal aspect of the first MTPJ;

(vi) be able to walk household distances (>50 meters) without the aid of a walker, crutches or cane;

(vii) be willing to attend the Health Sciences Clinic at La Trobe University (Melbourne, Victoria) on two occasions and have their foot x-rayed;

(viii) be willing to not receive additional interventions (such as physical therapy, foot orthoses, shoe modifications, intra-articular injections, or surgery) for the first MTPJ pain during the course of the study;

(ix) be willing to discontinue taking all medications to relieve pain at their first MTPJ (analgesics and non-steroidal anti-inflammatory medications [NSAIDs], except paracetamol up to 4 g/day) for at least 14 days prior to the baseline assessment and during the study period. Participants who do take paracetamol for first MTPJ pain need to discontinue its use at least 24 hours prior to the baseline assessment and follow-up assessments at 4, 8 and 12 weeks.

Exclusion criteria for participants in this study will be:

(i) pregnancy;

(ii) previous surgery on the first MTPJ;

(iii) significant deformity of the first MTPJ including hallux valgus (grade of 3 or 4 scored using the Manchester Scale) [42,43];

(iv) presence of one or more conditions within the foot or ankle, in the opinion of the investigators, could confound pain and functional assessments of the first MTPJ, such as metatarsalgia, plantar fasciitis, pre-dislocation syndrome, Achilles tendinopathy, degenerative joint disease (other than the first MTPJ);

(v) presence of any systemic inflammatory condition, such as inflammatory arthritis, rheumatoid arthritis, ankylosing spondylitis, psoriatic arthritis, reactive arthritis, septic arthritis, acute pseudogout, gout or any other connective tissue disease;

(vi) any medical condition that, in the opinion of the investigators, makes the participant unsuitable for inclusion (e.g., severe progressive chronic disease, malignancy, clinically important pain in a part of the musculoskeletal system other than the first MTPJ, or fibromyalgia);

(vii) cognitive impairment (defined as a score of <7 on the Short Portable Mental Status Questionnaire) [44];

(viii) intra-articular injections into the first MTPJ in the previous 6 months;

(ix) currently wearing contoured foot orthoses (although flat insoles will be permitted);

(x) currently wearing specialised footwear (footwear that has been custom-made or 'prescribed’ by a health-care practitioner);

(xi) currently wearing shoes that would not be able to accommodate a foot orthosis, or;

(xii) older people with a history of recurrent falls (defined as 2 or more falls in the previous 12 months), as there is some evidence that rocker-sole shoes may have short-term detrimental effects on balance [45].

Participants will be recruited by advertisements placed in local newspapers, magazines, and social media, by posters placed in healthcare facilities, senior citizens’ centres, retirement villages and mail-out advertisements to health care practitioners in Melbourne. Although the recruitment strategy will focus on older people (due to their increased prevalence of OA), our inclusion criteria are not restricted to those aged over 65 years, as in our recently completed RCT of visco-supplementation for first MTPJ OA [17], the age range of participants with first MTPJ pain and radiographically confirmed first MTPJ OA was 22 to 81 years, with a mean age of 54 years, indicating that the condition is also prevalent in younger and middle-aged people.

### Rocker-sole footwear group

**Figure 2:**
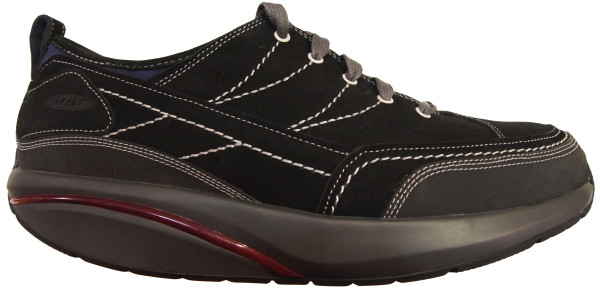
**MBT**^**® **^**Matwa footwear.**

The rocker-sole footwear group will be provided with a pair of appropriately-sized rocker-sole shoes (MBT® Matwa, Masai Barefoot Technology, Switzerland). This shoe is characterised by a rounded sole in the antero-posterior direction and a soft cushioned heel. Across the full size range, the radius of curvature of the MBT is on average 33 cm overall, 18 cm at the forefoot, 43 cm at the midfoot, and 11 cm at the heel [46]. See Figure 2.

### Prefabricated foot orthoses group

**Figure 3:**
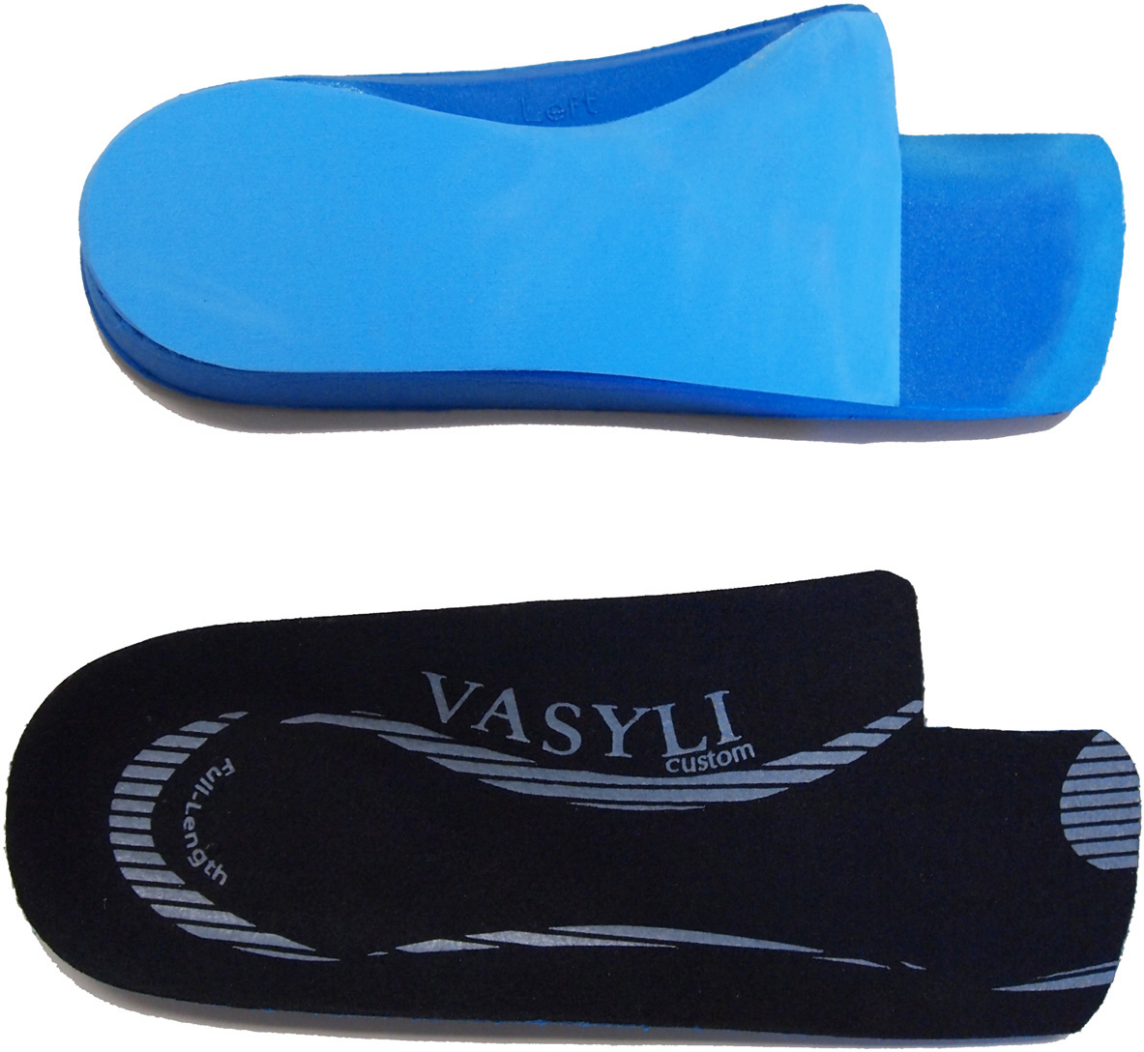
**Prefabricated foot orthoses to be used in the trial.** Top: plantar surface of left foot orthosis. Bottom: dorsal surface of right foot orthosis.

The prefabricated foot orthoses group will receive a pair of foot orthoses (Vasyli Customs – medium density, Vasyli Medical, Queensland, Australia) that will be modified using a similar approach to that described by Welsh *et al*. [35]. All orthoses will be full-length, but modified by adding a first ray cut-out and trimming the distal edge to the level of the 2nd to 5th toe sulci. In participants with pronated feet, full length 4-degree medial (varus) wedges (Formthotics, Foot Science International, Christchurch, New Zealand) will be applied to the underside of the foot orthoses (see section on the Foot Posture Index below). Those who do not have pronated feet will have open cell polyurethane foam applied to the plantar aspect of the rearfoot. See Figure 3.

### Baseline assessments

#### *Demographic factors, major medical conditions, health-related quality of life and anthropometrics*

Demographic factors (such as age, sex, education and income), major medical conditions and number of medications currently prescribed, will be obtained via a structured questionnaire. Height and weight will be measured using a stadiometer and digital scales, respectively, and body mass index will be calculated as weight (kg)/height (m)^2^. In addition, two questionnaires will be included at baseline as potential predictors of treatment outcome: the Pain Catastrophizing Scale, a questionnaire containing 13 items reflecting elevated negative cognitive responses to pain [47], and relevant questions from the Monitor Orthopaedic Shoes questionnaire [48], which assesses participants’ expectations of footwear-related interventions. Footwear will also be assessed using selected items from the Footwear Assessment Tool [49].

#### *Clinical assessment of first MTPJ OA features*

The following clinical observations will be used to characterise the clinical features of first MTPJ OA in the sample. The reliability of these observations has been reported in detail previously (kappa values from 0.41 to 1.00 [41]).

(i) dorsal exostosis: the presence of a definite dorsal bony exostosis will be determined via visual observation of the first MTPJ;

(ii) effusion: a palpable joint effusion around the dorsal, dorso-medial and dorso-lateral regions will be determined using manual palpation. An effusion will be classified as present if the area is compressible and displacement of fluid noted;

(iii) pain during motion: the examiner will grasp the proximal phalanx of the hallux and dorsiflex the digit until movement is no longer possible. This process will be repeated 5 times with a positive test result concluded if end range of motion produces pain in at least 2 of the test trials;

(iv) hard end-feel: the examiner will grasp the proximal phalanx and dorsiflex the hallux until movement is no longer possible. A positive test result will be documented if a hard osseous end-feel is determined as opposed to a gradual end-feel of joint motion [50];

(v) crepitus during dorsiflexion: the examiner will apply a compressive force while moving the joint through its full range of dorsiflexion motion. This process will be repeated 5 times with a positive test result documented if a grating or cracking sensation occurs during at least 2 of the test trials [51];

(vi) first MTPJ dorsiflexion range of motion: the passive, non-weightbearing, dorsiflexion range of motion of the first MTPJ will be measured in accordance to the procedure described by Hopson *et al.*[52]. The first metatarsal and proximal phalanx of the hallux will initially be bisected in the sagittal plane. A dorsiflexion force will be applied to the hallux until end range of motion is reached, allowing the first ray to maximally plantarflex. The angle between the two lines will then be measured via a hand held goniometer. This measurement technique has been shown to be highly reliable in people with first MTPJ pain (intra-class correlation coefficient of 0.95 [41]). The assessment will be performed twice and the average score will be documented.

#### *Foot Posture Index (FPI)*

To help determine the amount of rearfoot control to be added to the orthoses in the prefabricated foot orthoses group, the FPI will be documented. The FPI is a valid and reliable clinical assessment tool [53] which consists of six specific criteria: talar head palpation, supralateral and infralateral malleolar curvature, calcaneal frontal plane position, prominence in the region of the talonavicular joint, medial arch height and abduction and adduction of the forefoot on the rearfoot. Each FPI criterion is scored on a 5-point scale (range, -2 to +2). The six scores obtained are then summated to give an overall score of foot posture. The summated score has the potential to range from -12 (highly supinated) to +12 (highly pronated). The normal FPI (defined as ± 1 standard deviation from the mean) has been reported to be +1 to +7 [54]. Therefore, participants who score greater than 7 will be deemed to have pronated foot posture, and will have full length medial wedges applied to the plantar aspect of the foot orthoses. The magnitude of the wedge angulation will be increased until there is a reduction in the FPI score of at least 2 points [35]. Those with an FPI score less than 7 will have open cell polyurethane foam applied to the plantar aspect of the rearfoot.

#### *Three-dimensional foot scanning*

The FotoScan 3D foot scanner (Precision 3D Ltd, Weston-super-mare, UK) will be used to obtain fully weight bearing scans of both feet. Participants will be instructed to stand relaxed with their feet approximately shoulder width apart and their hands clasped lightly in front of them or gently resting on the support rail if required. The FotoScan 3D device uses a fixed system of cameras and projectors to obtain digital images of the foot, which are then automatically converted to 3D models. According to the manufacturer, the scans obtained with this system are accurate to within less than half a millimetre.

#### *Radiographic assessment of OA*

The presence of radiographic OA will not be an inclusion criterion, as in clinical practice, footwear interventions are often provided on the basis of clinical findings without confirmation of radiographic changes. However, to document the radiographic severity of OA, weightbearing dorso-plantar and lateral radiographic projections will be obtained from the most symptomatic foot (or in the case of equivalent bilateral symptoms, the right foot), with the participant standing in a relaxed bipedal stance position. All x-rays will be taken by the same medical imaging department using a Shimadzu UD150LRII 50 kw/30 kHz Generator and 0.6/1.2 P18DE-80S high speed x-ray tube from a ceiling suspended tube mount. AGFA MD40 CR digital phosphor plates in a 24 cm × 30 cm cassette will be used. The tube will be angled 90 degrees and centred at the base of the third metatarsal, with the film focus distance set at 100 cm. All radiographs will be examined by HBM, SEM and JMT, to determine the presence of OA, using a radiographic atlas of foot OA developed by our group [55,56]. The reliability of assessments using this atlas have been reported in detail previously (kappa values greater than 0.76 [55]).

#### *Treatment preference and credibility/expectation*

In open randomised trials of musculoskeletal disorders, it has been demonstrated that participants who are allocated to their preferred treatment achieve better outcomes than those who do not receive their preferred treatment, despite the randomisation process resulting in equivalent baseline outcome measure scores [57]. To address this issue, participants will be asked if they have a preference for either treatment (documented as rocker-sole shoes, prefabricated foot orthoses or no preference), although their response will not influence their randomised group allocation [58]. This approach conserves all the advantages of a fully randomised design but enables the interaction between preference and outcomes to be quantified [59]. A related issue is treatment credibility (participants’ beliefs about the logic underpinning the intervention) and treatment expectancy (participants’ perceptions of how much they may benefit) [60]. To quantify this, the Credibility/Expectancy Questionnaire (CEQ) [61] will be administered after randomisation. The CEQ consists of six items: three related to credibility and three related to expectancy. For each item, participants will be asked to rate the credibility of the treatment and their expectations on a 9-point Likert scale. High scores on the scale indicate that the participant considers the treatment to be credible and expects the treatment to be effective. The CEQ has been shown to have good internal consistency and test-retest reliability [61], and has recently been used to assess the credibility of sham dry needling [62] and sham foot orthoses [63] used in clinical trials.

#### *Biomechanical evaluation*

Because the proposed mechanism of action of the rocker-sole shoes is a change in biomechanical function of the lower limb, two approaches will be used to evaluate their effects at the baseline appointment. First, spatio-temporal parameters and three-dimensional motion of the thigh and lower leg during gait will be recorded using a wireless, wearable sensor motion analysis system (LEGSys™, Biosensics, Boston, MA, USA). This system consists of accelerometers and gyroscopes attached with Velcro™ straps to each lower leg and thigh. The method for calculation of the spatio-temporal parameters of gait is described in detail elsewhere [64]. To summarise, the gait phases are determined from the precise events of heel-strike (initial foot contact) until toe-off (terminal foot contact). These events are extracted from gyroscopes attached to each shank through a local minimal peak detection scheme. Based on each participant’s height and using a biomechanical model, spatial parameters (i.e. stride length and stride velocity) and kinematics (knee and hip) are estimated by integration of the angular rate of rotation of the thigh and shank relative to the waist sensor. Gait analysis with this system has been validated in healthy controls [64], older people [65], and patients with hip osteoarthritis [66]. After a familiarisation period, participants will complete 4 walking trials for each footwear condition over an 8 metre distance. To exclude the effect of acceleration and deceleration steps, only the middle four steps from each trial will be included for analysis. An average recording will be determined from the 16 steps (4 steps from 4 trials) for each condition. Second, plantar pressures (specifically, peak pressure under the hallux, first MTPJ and lesser MTPJs) will be measured with the in-shoe Pedar® system (Novel GmbH, Munich, Germany), a reliable, valid and accurate measure of in-shoe pressure [67-69]. The Pedar® insoles are approximately 2 mm thick and consist of 99 capacitive pressure sensors, arranged in grid alignment. Plantar pressure data will be sampled at a frequency of 50 Hz. Both groups will undergo the same biomechanical assessment. However, for rocker-sole shoe group, comparisons will be made between their own shoes and the rocker-sole shoes, while in the prefabricated foot orthoses group, comparisons will be made when wearing their own shoes (with and without the prefabricated orthoses).

### Outcome measures

Primary and secondary outcome measures will be collected at baseline and 4, 8 and 12 weeks. These time points have been selected as 4 weeks is considered the earliest time of greatest effect and 12 weeks is considered to be a standard clinical follow-up time [17,70]. To avoid over-testing and to minimise the risk of Type I error associated with serial measurements, statistical analysis of the effectiveness of the interventions will specifically focus on the change in primary outcome measures between baseline and 12 weeks [71,72].

The primary outcome measure will be the foot pain domain of the Foot Health Status Questionnaire (FHSQ) [73]. The FHSQ is a foot-specific health-related quality of life outcome measure consisting of 13 questions that assess four domains of foot health including pain, function, footwear and general foot health. Questions within each domain are scored using a Likert response format, with an output score produced ranging from 0 to 100, with a score of 100 indicating optimum foot health and a score of 0 indicating very poor foot health. The FHSQ has been shown to have a high degree of internal consistency (Cronbach's α = 0.88) and test-retest reliability (intra-class correlation coefficient = 0.86 [73]), and has been used in several RCTs by our group [17,62,74]. A recent review recommended the use of the FHSQ in clinical trials of rheumatological foot disorders [75]. Participants treated for bilateral symptoms will be asked to describe symptoms of their most painful foot. If both feet are equally painful, the right foot will be selected as the index foot.

Secondary outcome measures will include:

(i) the function domain of the FHSQ, measured at baseline, 4, 8 and 12 weeks;

(ii) the Foot Function Index - Revised (Short Form) [76], measured at baseline and 12 weeks;

(iii) severity of pain at the first MTPJ while walking over a flat surface and during rest over the last week (each via a 100 mm visual analog scale [VAS]), measured at baseline, 4, 8 and 12 weeks;

(iv) duration and severity of stiffness at the first MTPJ after first awakening in the morning, during the last week (via a 100 mm VAS), measured at baseline, 4, 8 and 12 weeks;

(v) severity of stiffness after sitting, lying, or resting later in the day, during the last week (via a 100 mm VAS), measured at baseline, 4, 8 and 12 weeks;

(vi) global change in symptoms using a 15-point Likert scale (The responses will range from "A very great deal better" to "A very great deal worse") measured at 12 weeks;

(vii) health status (using the Short-Form-12 Version 2 questionnaire) [77], measured at baseline and 12 weeks;

(viii) use of paracetamol rescue medication (number of participants and mean consumption) and co-interventions to relieve pain at the first MTPJ, documented with a monthly diary throughout the 12 week study period;

(ix) the frequency and type of self-reported adverse events (such as falls and development of new pain in other body regions), collected at 4 weekly intervals throughout the 12 week study period;

(x) the Incidental and Planned Activity Questionnaire, a self-report questionnaire that covers the frequency and duration of several levels of planned and incidental physical activity [78], measured at baseline and 12 weeks.

To maximise response to the postal questionnaire outcome measures, we will send a postcard reminder after one week to non-responders, and then follow-up with up to three attempted contacts by telephone and/or email over a two week period.

### Sample size

The sample size for the study has been determined using an *a priori* power analysis based on the primary outcome measure: the pain domain of the FHSQ [73]. We have previously determined that the minimal important difference for this measure in people with foot pain is 13 points [79]. Using a standard deviation of 19 (derived from our recent trial [17]), a power level of 0.8, alpha level of 0.05 and accounting for a drop-out rate of 15%, a sample size of 80 participants (i.e. approximately 40 per group) will be required. We have conservatively ignored the extra precision provided by covariate analysis when estimating the sample size, and have conservatively selected a drop-out rate at the higher end of previous trials of specialised footwear and/or orthoses for lower limb OA (between 2 and 15%) [30,80-82].

### Evaluation of adherence

Adherence to the intervention in both groups will be documented at 4, 8 and 12 weeks. Participants will provide information concerning the number of hours per day and number of days they have worn their rocker-sole shoes or prefabricated foot orthoses during the previous 4 weeks. To minimise participant burden, adherence will be documented on the day with recall over the previous 4 weeks, rather than by daily diary entries.

### Complications and adverse events

Complications and adverse events associated with the intervention are unlikely. However, the questionnaires at the 4, 8 and 12 week follow-ups will provide participants with an opportunity to report any difficulties they have with the interventions, and all adverse events will be reported in the final manuscript.

### Statistical analysis

Statistical analysis will be undertaken using SPSS® version 21.0 (IBM Corp, NY, USA) using the intention-to-treat principle for all randomised participants [38]. The exception to this will be the safety outcome measures which will be analysed as treated. In participants with bilateral symptoms, the most painful foot will be analysed. Multiple imputation will be used to replace any missing data using five iterations, with age, baseline scores, and group allocation as predictors [83]. The exception will be for the variables use of rescue medication and co-interventions where no data substitution will be applied. Standard tests to assess continuous data for normal distribution will be used and transformation carried out if required. Differences in the primary and secondary outcome measures between the two groups will be compared. Continuously-scored outcome measures will be analysed using analysis of covariance with baseline scores and intervention group entered as independent variables [84]. Ordinal scaled data will be analysed using non-parametric tests. Dichotomously-scored outcome measures will be compared using relative risk, risk difference and number needed to treat. For the gait analysis component of the study, differences in biomechanical variables will be compared using paired *t*-tests and effect sizes (or in the case of non-normally distributed data, medians will be compared using Mann–Whitney U tests).

## Discussion

The objective of this trial is to compare the effectiveness of rocker-sole footwear and individualised prefabricated foot orthoses in reducing pain associated with OA of the first MTPJ. This will be the third clinical trial undertaken for this condition and the first to focus on footwear-related interventions. Our recent systematic review of interventions for first MTPJ OA [15] revealed only one low quality randomised trial evaluating the effectiveness of two different physical therapy programs in 20 participants [16], and more recently, our group completed an RCT which found that intra-articular viscosupplementation (hylan G-F20) was no more effective than a placebo (sterile saline) [17]. As such, the trial will provide novel and clinically relevant information to inform the non-surgical management of this common and disabling condition.

The underlying biomechanical rationale for the use of mechanical interventions for first MTPJ OA is that the condition results from the inability of the first metatarsal to plantarflex during propulsion to allow the proximal phalanx to dorsally rotate on the first metatarsal head, thereby resulting in dorsal joint compression of the first MTPJ [12,31]. However, the two interventions to be compared in this trial aim to address this biomechanical deficit in different ways. Rocker-sole footwear is thought to reduce the need for first MTPJ dorsiflexion during propulsion, by enabling the body’s centre of mass to “rollover” the base of support, thereby decreasing sagittal plane motion of the ankle [19-22], forefoot [19] and first MTPJ [24], and reducing forefoot plantar pressures [22,23]. In contrast, foot orthoses with a first ray cut-out, forefoot extension and medial wedging aim to limit rearfoot pronation and facilitate first ray plantarflexion during propulsion, thereby allowing the proximal phalanx to rotate on the first metatarsal head to achieve sufficient first MTPJ dorsiflexion [31]. The available evidence addressing this proposed mechanism of action of foot orthoses, however, is equivocal. While two studies have reported increases in first MTPJ dorsiflexion with orthoses placed under the foot in static stance [85,86], one study reported no change in first MTPJ dorsiflexion [87]. Gait studies have been similarly inconsistent, with two studies reporting a *decrease* in first MTPJ dorsiflexion (with medial wedging [88] and orthoses [34]) and one study reporting no change [35].

The prefabricated orthoses in this study will be modified in a similar manner to the protocol described by Welsh *et al*. [35]. However, there are two key differences in the inclusion criteria between our two studies. In the Welsh *et al*. study, participants were excluded if they had an FPI score <4 or exhibited less than 40 degrees of non-weightbearing first MTPJ dorsiflexion. This approach is based on three assumptions: (i) that people with first MTPJ OA are more likely to have pronated feet; (ii) that control of rearfoot pronation is an important goal of treatment for this condition, and; (iii) that individuals with less than 40 degrees of non-weightbearing first MTPJ dorsiflexion would not benefit from the orthoses. Participants in our trial will not be excluded on the basis of foot posture, as our recent systematic review did not find pronated foot posture to be strongly associated with this condition [12]. Furthermore, the gait analysis component of the Welsh *et al*. study found no differences in rearfoot motion with the orthoses, suggesting that control of rearfoot pronation did not contribute to the observed improvement in symptoms. Finally, participants in our trial will be required to have less than 64 degrees of first MTPJ dorsiflexion (as we have shown that this cut-off is associated with radiographic OA in the first MTPJ [41]), but we will not exclude any participants on the basis of having insufficient range of motion in the first MTPJ. It could be argued that individuals with very limited first MTPJ range of motion have little to gain from prefabricated foot orthoses with a modification (a first ray cut-out) that, theoretically, facilitates motion at the first MTPJ by allowing the first ray to plantarflex. However, this mechanism is yet to be adequately demonstrated, and it is possible that the orthoses could achieve beneficial therapeutic effects through other mechanisms, such as reducing peak pressure under the hallux and first MTPJ [85].

The gait analysis component of the study will provide useful insights into the possible mechanism of action of the interventions. The wearable sensor motion analysis system (LEGSys™, Biosensics, Boston, MA, USA) provides high resolution temporal and kinematic data, without the laboratory constraints and data processing requirements of traditional motion analysis systems [89]. The use of this system will help identify changes in lower limb function associated with the footwear and orthoses (particularly changes in sagittal plane motion of the shank and thigh), and whether these changes are related to improvement in symptoms. Similarly, the in-shoe plantar pressure system (Pedar®, Novel GmbH, Munich, Germany) will identify the extent to which the high peak pressures under the hallux frequently observed in individuals with first MTPJ OA [6,90] are mitigated by the interventions. It should be noted, however, that it is not possible to measure first MTPJ motion *directly* in this study, as this requires the permanent modification of the upper of the shoe to allow placement of reflective markers or electromagnetic motion sensors on the foot.

Participants will be reviewed at 4-weekly intervals up to 12 weeks, which is similar to previous footwear trials which have employed 8 week [91] or 12 week [29,30,92] follow-up periods, and longer than the 4 week physical therapy trial for first MTPJ OA by Shamus *et al*. [16]. Although our trial of viscosupplementation for first MTPJ OA followed participants for 24 weeks, the effect of treatment clearly plateaued at 12 weeks in both groups [17]. We are therefore confident that the 12 week duration of the trial is sufficient to detect differences between the groups, if they exist. Furthermore, 12 weeks is considered to be a standard clinical follow-up period in trials evaluating the effects of interventions for OA [70]. The trial will incorporate 4 repeated measurements for the primary outcome measure (baseline, 4 weeks, 8 weeks and 12 weeks) to provide insights into the trajectory of any improvements in symptoms. However, to address the issue of multiple testing of serial measurements [71,72], we have pre-specified 12 weeks as the primary end-point, and no statistical comparisons of the 4 and 8 week scores will be undertaken.

There are three key limitations of the study design. Firstly, this is not a *controlled* trial, as both groups will receive an active intervention that has the potential to be therapeutically beneficial. This was an ethical requirement of our institution, as our original proposal to include “sham” orthoses as the comparator [63] was considered by the ethics committee to be withholding “usual” care. Because usual care is not well documented for this condition, we selected what we believe to be a simple, commonly-used treatment (prefabricated foot orthoses) with some evidence to support it, albeit it relatively low quality (case series evidence) [35]. This change in protocol unavoidably reduces the methodological rigour of the trial, but also increases its external validity.

Secondly, due to the nature of the interventions, research staff cannot be blinded to group allocation. However, follow-up assessment of outcome measures will be via self-completion questionnaires returned by mail, and research staff entering the data and conducting the analyses will be blinded to group allocation. Blinding participants in relation to the two treatments being offered was considered (i.e. by referring to them as “two footwear-related interventions” rather than specifying the rocker-soled shoes and foot orthoses). However, the inherent risk of this approach is that some participants may not be prepared to the wear the rocker-soled shoes for aesthetic reasons (and some could withdraw from the study immediately after being randomised for this reason). On balance, we considered that the reduction in methodological rigour created by poor adherence and differential drop-out was probably greater than that created by lack of blinding; hence our decision to openly advise participants as to the two treatments being compared.

A third, related issue is that because we will openly disclose the two treatments during the recruitment process, there is some risk of “resentful demoralisation” in participants who are allocated to the prefabricated foot orthoses group. That is, some participants who are allocated to receive the prefabricated foot orthoses may be resentful of not receiving the more expensive and (more visually substantial) rocker-soled shoe intervention, which may affect their adherence to the tasks required of them in the study and systematically influence their responses to the outcome measure questionnaires [58]. To address this, we will document both participant preference prior to randomisation and participants’ perceptions of treatment credibility/expectation after randomisation. This approach will not *prevent* resentful demoralisation, but will enable post-randomisation effects of treatment preference to be quantified, and if necessary, factored into the analysis as a prognostic variable.

### Trial status

This study will be the first randomised trial to compare the effectiveness of rocker-sole footwear and individualised prefabricated foot orthoses in reducing pain associated with osteoarthritis of the first MTPJ, and only the third randomised trial conducted for this common and disabling condition. Recruitment of participants will commence in February 2014 and final results are expected to be available in June 2015.

## Competing interests

The authors declare that they have no competing interests.

## Authors’ contributions

HBM, SEM and PL conceived the idea and obtained funding for the study. HBM, SEM and PL designed the trial protocol with input from JMT, MA and ER. HBM drafted the manuscript with input from SEM, PL, JMT, MA and ER. All authors have read and approved the final manuscript.

## Pre-publication history

The pre-publication history for this paper can be accessed here:

http://www.biomedcentral.com/1471-2474/15/86/prepub
